# Enhancing timeliness of drug overdose mortality surveillance: A machine learning approach

**DOI:** 10.1371/journal.pone.0223318

**Published:** 2019-10-16

**Authors:** Patrick J. Ward, Peter J. Rock, Svetla Slavova, April M. Young, Terry L. Bunn, Ramakanth Kavuluru

**Affiliations:** 1 Kentucky Injury Prevention and Research Center, College of Public Health, University of Kentucky, Lexington, Kentucky, United States of America; 2 Department of Epidemiology, College of Public Health, University of Kentucky, Lexington, Kentucky, United States of America; 3 Department of Biostatistics, College of Public Health, University of Kentucky, Lexington, Kentucky, United States of America; 4 Center on Drug and Alcohol Research, College of Medicine, University of Kentucky, Lexington, Kentucky, United States of America; 5 Department of Preventive Medicine and Environmental Health, College of Public Health, University of Kentucky, Lexington, Kentucky, United States of America; 6 Department of Computer Science, College of Engineering, University of Kentucky, Lexington, Kentucky, United States of America; 7 Division of Biomedical Informatics, Department of Internal Medicine, College of Medicine, University of Kentucky, Lexington, Kentucky, United States of America; Politechnika Krakowska im Tadeusza Kosciuszki, POLAND

## Abstract

**Background:**

Timely data is key to effective public health responses to epidemics. Drug overdose deaths are identified in surveillance systems through ICD-10 codes present on death certificates. ICD-10 coding takes time, but free-text information is available on death certificates prior to ICD-10 coding. The objective of this study was to develop a machine learning method to classify free-text death certificates as drug overdoses to provide faster drug overdose mortality surveillance.

**Methods:**

Using 2017–2018 Kentucky death certificate data, free-text fields were tokenized and features were created from these tokens using natural language processing (NLP). Word, bigram, and trigram features were created as well as features indicating the part-of-speech of each word. These features were then used to train machine learning classifiers on 2017 data. The resulting models were tested on 2018 Kentucky data and compared to a simple rule-based classification approach. Documented code for this method is available for reuse and extensions: https://github.com/pjward5656/dcnlp.

**Results:**

The top scoring machine learning model achieved 0.96 positive predictive value (PPV) and 0.98 sensitivity for an F-score of 0.97 in identification of fatal drug overdoses on test data. This machine learning model achieved significantly higher performance for sensitivity (*p*<0.001) than the rule-based approach. Additional feature engineering may improve the model’s prediction. This model can be deployed on death certificates as soon as the free-text is available, eliminating the time needed to code the death certificates.

**Conclusion:**

Machine learning using natural language processing is a relatively new approach in the context of surveillance of health conditions. This method presents an accessible application of machine learning that improves the timeliness of drug overdose mortality surveillance. As such, it can be employed to inform public health responses to the drug overdose epidemic in near-real time as opposed to several weeks following events.

## Introduction

Death certificates (DCs) are the primary source for state and local drug overdose (OD) mortality surveillance and are currently the only nationwide source [1]. DCs provide information about decedents (including demographic information, residence, and place of death), cause and manner of death, and substance(s) involved in an OD that are important to developing drug OD prevention programs and policies [2, 3]. In order to design and implement effective public health interventions, this information must be available to public health practitioners in a timely manner.

In the case of a suspected drug OD death, a coroner or a medical examiner serving the jurisdiction where the death occurred determines the cause-of-death and completes a DC [4, 5]. The DC is then filed (electronically or as a paper copy) with the state office of vital statistics (OVS). An electronic record with selected DC fields, including the free text information for the cause-of-death [6], is transmitted to the National Center for Health Statistics (NCHS) and coded according to the guidelines of the International Classification of Diseases, Tenth Revision (ICD-10) to allow standardized classification of the causes of death [7–10]. A copy of the ICD-10-coded record, containing one underlying cause-of-death (UCOD) and up to 20 supplementary causes-of-death, is sent back to the state OVS to be used for epidemiological analysis. There is a significant time lag between the day of death and the day when an ICD-10-coded DC record is available for identification of a drug OD death (the consensus definition for drug OD mortality surveillance is based on the UCOD code in the range X40-X44, X60-X64, X85, or Y10-Y14 [11, 12]). Spencer et al. reported that only 37.8% of the drug OD death certificates are available to NCHS by 13 weeks (vs. 83.9% for overall deaths), mostly due to delays in DC completion related to required forensic toxicology analysis [13]. Additional time lag is acquired at the NCHS as about two-thirds of the deaths with an UCOD of drug OD are coded manually, compared to one-fifth of all-cause deaths [14].

### Motivation

**Fig 1:**
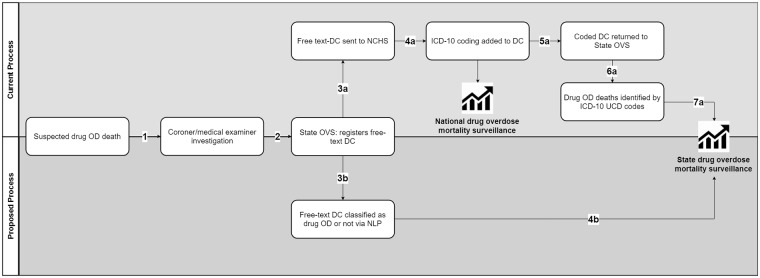
Certification, registration, and analysis of drug overdose deaths.

Understanding the critical role of surveillance data to inform prevention and response to the opioid epidemic, the Centers for Disease Control and Prevention (CDC) provided dedicated funding to states to build capacity for more timely and comprehensive opioid OD surveillance data [15]. This paper examines the feasibility of using natural language processing (NLP) and machine-learning (ML) methods to identify potential OD deaths from free-text DC fields, allowing for the identification of potential drug OD deaths (and the initiation of gathering of additional medicolegal data for these cases) before the DC records are sent to the NCHS for ICD-10 coding. Fig 1 displays the overall workflow of the death investigation and public health surveillance approach; the proposed method eliminates the time lag associated with steps 3a-7a, replacing these with steps 3b and 4b. We chose NLP and ML as the methodological base given they (a) provide an intuitive mapping from free text fields to categories using classification techniques and (b) are generally more accurate than rule-based systems in data rich settings. Our main goal is to build a practical computational solution that can be employed by epidemiologists in public health agencies for near real time OD mortality surveillance with reasonably high accuracy. Hence, all the code used in this effort is made publicly available: https://github.com/pjward5656/dcnlp.

### NLP and ML background

Text is ubiquitous in healthcare and biomedicine coming from different sources including biomedical literature (journal articles, conference abstracts), clinical notes (e.g., discharge summaries and pathology reports), and social media text (e.g., Twitter, Reddit, and specialized forums such as the Cancer Survivors Network). Text classification methods from NLP and ML have been shown to play a critical role in health and biomedical applications especially when structured sources do not fully capture all the information necessary. The NLP component deals with extracting interesting “features” (independent variables) based on simple n-grams (typically words and two-to-three word phrases) and more involved syntactic constructs such as parts-of-speech for each word (e.g. noun, adjective) and constituency and dependency parse trees that represent inter-word grammatical relations within each sentence. The ML component then learns a model with these features as independent variables and the category as the outcome variable using, typically, hand-coded training data.

A basic text classification problem typically deals with an outcome variable that is **binary**. For example, based on a pathology report can we identify whether a cancer case is reportable or not? [16]. There could also be cases where the problem is **multiclass**, where one of more than two categories ought to be chosen. For instance, in cancer registries, certified tumor registrars read pathology reports to code major sites from a list of dozens of such sites and this task can be expedited using automated methods [17]. A variant of the multiclass problem is **ordinal** classification, where the categories are ordered in some manner specific to the task. Although one class is chosen in ordinal models, errors are counted differently based on how far away the prediction is from the true category. As an example, in psychiatry a recent shared application [18] dealt with assigning symptom severity categories (*absent*, *mild*, *moderate*, and *severe*) based on content in a psychiatric evaluation note. Finally, **multilabel** classification handles scenarios where more than one category is typically assigned to each input instance. A use-case is when coders assign multiple diagnosis codes [19] to electronic medical records for every patient visit. For elaborate details of specific applications of NLP in biomedicine, please refer to broad reviews [20, 21]. The problem at hand in this current effort is binary classification to identify deaths due to OD based on DC text.

## Methods

### Data

This research was conducted as part of the University of Kentucky’s Enhanced State Opioid Overdose Surveillance project, which has been reviewed and approved by the Institutional Review Board at the University of Kentucky. The Kentucky Injury Prevention and Research Center (KIPRC), as bona fide agent of the Kentucky Department for Public Health, receives weekly extracts of DCs to perform injury surveillance. Coded DCs for years 2017–2018 as of November 1^st^, 2018 (n = 84,142), were used for this analysis. The ML process requires that the data is split into training and testing sets; the coded 2017 DCs (n = 48,016) were used as training data and the coded 2018 DCs (n = 36,126) were used as testing data. In total, 2,478 (2.9%) of the DCs were coded as OD deaths based on the ICD-10 code assigned by NCHS in the UCOD field [22]. These cases were treated as the “true positive” cases when training and testing the ML algorithm.

### Task

The task for the ML algorithm in this paper is to take an un-coded DC and classify it as an “OD death” or “not OD death” using free-text fields. To automate this task free-text fields on the DC were used to create features for a classifier. ML algorithms require feature vectors to train the model. In this analysis, a feature is a binary [0,1] variable created from an aspect of the free-text present on the DC, and a feature vector is *z*-dimensional vector of features where *z* is the total number of features.

### Feature engineering

DCs in Kentucky are certified by county coroners or physicians depending on the manner of death. The certifier completes the cause-of-death section, consisting of an “immediate cause” field (“line a”) followed by three sequential fields indicating conditions that were “leading to the cause listed in line a” [14]. In addition, the certifier may also complete two other sections: 1) “other significant conditions contributing (SCC) to death but not resulting in the underlying cause” and 2) “describe how the injury occurred.” The latter is only completed if the death resulted from an injury.

These free-text fields (used by the NCHS to assign ICD-10 codes for underlying and contributing causes-of-death) were used to create features for the ML algorithm. Two different field combinations were examined for this task: 1) all three free-text sections of the death certificate and 2) the cause-of-death section and the description of injury section. The latter option was considered as ODs and substance use may not cause death but may contribute to other types of morbidity that later cause death. Excluding the SCC section may result in better separation between OD deaths and deaths that involved substance use or previous ODs.

Certifiers in Kentucky typically do not write in full sentences using grammatically or syntactically correct language when completing the DC. The text is often concise. Table 1 shows an example of a typical OD DC. Due to these considerations, to simplify the task the fields in the training data were combined into one free-text field. All punctuation was removed from this field. Using the scikit-learn [23] library in Python, this field was tokenized into individual words, bigrams (adjacent two-word sequences), and trigrams (adjacent three-word sequences), excluding stop words (“the”, “an”, “and”, etc.). All free-text in the data exists in fully capitalized form, and the capitalized tokens were used, unaltered, for the analysis. Any token appearing less than five times was discarded. The 2017 DC data including the SCC field contained 2,184 unique words and 11,261 bi/tri-grams. When excluding the SCC field, the word and bi/tri-gram list decreased to 1,820 and 8,029, respectively. Features for each of these words and phrases were then created for the model; a feature was given the value of 1 if the word/phrase it represents appeared in the text and a value of 0 otherwise. To illustrate, a feature representing the bigram “acute cocaine” would be 1 for the DC in Table 1 while a feature for the word “poisoning” would be 0.

**Table 1:** Example death certificate free text.

Field	Text
Immediate cause-of-death	ACUTE COCAINE TOXICITY
Due to (or as a consequence of)	
Due to (or as a consequence of)	
Due to (or as a consequence of)	
Significant conditions contributing	HYPERTENSIVE CARDIOVASCULAR DISEASE, OBESITY
Description of injury	ACCIDENTAL OVERDOSE

### Classifiers

**Fig 2:**
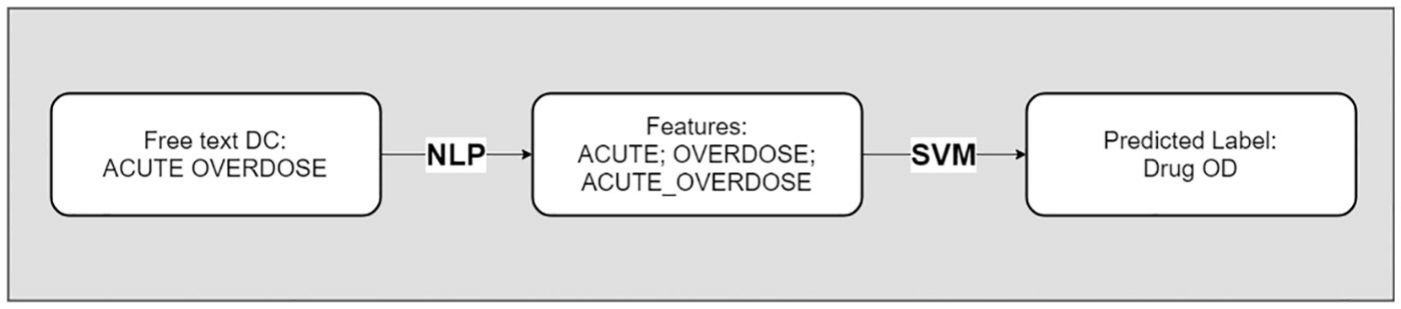
Example analytic pipeline.

Several classification methods were considered and examined for this task. Linear support vector machines (SVM), random forests (RF), and multilayer perceptrons (MLP) were tested for this classification task. The SVM approach was selected due to both the low computational requirements of this method as well as its use in previous, similar classification tasks [24–26], while RFs and MLPs were examined as more complex nonlinear methods that may identify additional interaction features compared to the linear SVM. Fig 2 shows the overall process used to predict if a DC is an OD using only the free-text fields. While this classification task was implemented in the Python environment, the algorithms tested have well documented and easily programmable methods within the e1071 and CARET [27] packages in R, making this an accessible method.

### Training

The algorithms were trained on all coded 2017 DCs using 3 times repeated, stratified 10-fold cross validation within the scikit-learn Python library. Repeated cross validation was selected as it is recommended over other methods for general classification use [28]. Stratified cross validation was used so that each fold created during the procedure would have the same makeup of OD deaths and non-OD deaths that the entire dataset has (~97% non-OD, ~3% OD) [29]. The cost (regularization) hyperparameter of the linear SVM, the maximum depth, number of trees, and maximum number of features hyperparameters of the RF, and the hidden layer sizes and alpha (regularization) hyperparameters of the MLP were tuned based on F-score, the harmonic mean of positive predictive value (PPV) and sensitivity [30]. F-score was selected as the tuning metric due to the class imbalance in the data; tuning based on accuracy would bias the algorithm to correctly classifying non-OD cases, as ~97% of the data are not OD deaths. To tune the hyperparameters, potential values were initially selected. After training on these values, an additional search was performed around the value(s) that the previous step indicated was the best value(s) for the hyperparameter(s). This process was repeated subsequent times until the ideal hyperparameter value(s) was identified. After this, the entire training data was re-trained using the ideal value(s). This tuning approach was implemented as it is a straightforward grid-search method commonly used for tuning ML algorithms [31].

### Testing

After the learner was trained on the 2017 DC data it was deployed on the 2018 DC data. The same word/phrase features used in the 2017 DC data were created for the 2018 DC data. Both free-text field combination algorithms were tested.

### Rule-based method

A final rule-based classification method was also tested for this analysis on the 2018 DC data. This rule-based method scanned the free text of the DCs for 37 words or bigrams that were indicative of an OD death. These words/bigrams were selected from a review of OD DCs by epidemiologists with a combined 10 years of experience in OD surveillance. For this review, 2017 OD deaths were examined and common tokens identified. These words and bigrams are available in S1 Appendix. Any DC that contained one or more of the words/bigrams in this list were automatically classified as an OD death, and any death that did not contain a word/bigram in this list were classified as a non-OD death. This rule-based method is a simpler version of previously proposed text-matching methods [32], and acts as a baseline to compare to the more computationally costly ML models.

### Evaluation

Methods were evaluated based on their performance on the test data. To compare the results of the methods the F-score was calculated along with sensitivity and PPV. For this task PPV was considered the most important metric, as large numbers of false positives could be problematic when attempting to develop near real-time interventions for an OD outbreak. Two-proportion *z*-tests were performed to test for statistically significant differences between the sensitivity and PPV of the best performing ML model and rule-based method.

## Results

The model including all three sections had a total of 13,445 features while the model excluding the SCC section had a total of 9,849 features. Computing time for training the ML models was not significant; even when using stratified 10-fold, 3 times repeated cross validation the longest any model took to train was roughly ~3 hours on a Windows machine with 32 GB of RAM. Deploying the models on the 2018 data took seconds. The rule-based method does not involve any training and produced predictions instantly.

Table 2 displays the F-score, sensitivity, and PPV from the final methods when training the models with features constructed from all three sections and Table 3 displays the results when training the models with features from the cause-of-death and description of injury sections. All of the models were highly specific. Only ~3% of all deaths in the data were OD deaths and all of the ML models had high performance in identifying deaths that were not OD deaths. The ML models without the SCC section, however, all performed equally or higher than their counterpart ML models with the SCC section with regards to sensitivity, PPV, and F-score. The SVM model without the SCC section was the best performing model overall, with an F-score of 0.9695, and also achieved the highest PPV (0.9622), while the RF model without the SCC section achieved the highest sensitivity (0.9803). Table 4 displays the confusion matrix for the SVM excluding the SCC section.

**Table 2:** Final model results on test data, features from all death certificate sections.

Method	PPV	Sensitivity	F-score
SVM	0.9549	0.9748	0.9647
RF	0.9328	0.9748	0.9533
MLP	0.9518	0.9737	0.9626
Rule-based	0.9215	0.9265	0.9240

**Table 3:** Final model results on test data, features from cause-of-death and description of injury fields.

Method	PPV	Sensitivity	F-score
SVM	0.9622	0.9770	0.9695
RF	0.9531	0.9803	0.9665
MLP	0.9621	0.9737	0.9678
Rule-based	0.9504	0.9243	0.9372

**Table 4:** Confusion matrix, SVM model, features from cause-of-death and description of injury fields.

		UCOD Label		
		Drug OD	Not Drug OD	Totals
Predicted Label	Drug OD	891	35	926
	Not Drug OD	21	35,179	35,200
	Totals	912	35,214	36,126

The rule-based models did not perform as well as their counterpart ML models. The rule-based model excluding the SCC section had similar PPV to the ML models (0.9504), however this model had lower sensitivity than the ML models (0.9243). The rule-based model excluding the SCC section had a higher F-score than the rule based model including this section, similar to the results of the ML models. Comparing the best performing ML model (SVM, Table 3) to the best performing rule-based model (Rule-based, Table 3), two-proportion single-tailed *z*-tests show that the ML model has a significantly higher sensitivity (*p*<0.001) but no statistical increase for PPV (*p* = 0.13).

## Discussion

### Model performance

This paper presents an accessible method to quickly identify OD deaths from free-text DCs for rapid surveillance purposes. The performance of the ML algorithms developed were very high. The F-score of 0.9695 for the best performing model is comparable, and in some cases superior, to that of other models for cause-of-death classification in the literature [24–26, 33, 34], many of which were trained on larger datasets with more common causes of death. The ML models performed higher than their counterpart rule-based models, including significantly higher results for sensitivity, providing evidence for further increasing the use of ML in public health surveillance over more traditional methods.

Of the methods compared, the ML models that excluded the SCC section produced the best performing models, as the ML models that included all three sections had more false positives than their counterpart models. This is likely because features that identify an OD death when appearing in the description of injury section or the cause-of-death section have different meaning in the SCC section. For example, the bigram “drug overdose” clearly indicates the death was caused by an OD when present in the cause-of-death section, but when present in the SCC section may indicate that the individual had a previous OD event, but it did not directly lead to death. Including the SCC section results in a feature space that contains information that is not directly involved in causing death, leading to a slightly biased model. Further research should examine including the SCC section in a classifier to identify drug-related deaths, which are important for drug-related surveillance.

### Public health implications

DC data is the only national source for OD mortality surveillance [1] and as such is extremely important for understanding the opioid epidemic and developing responses to it. These data, however, comes at a non-trivial time lag [13, 14] that prohibit availability of actionable data until several weeks following the event of an OD death. The method proposed in the present study eliminates the time lag from transferring DCs between the state OVS and the NCHS and the time for ICD-10 coding by the NCHS, thus allowing state or local jurisdictions to process their own mortality data as soon as the DC is available in their database with free-text cause-of-death information. This provides more timely provisional counts for OD deaths, allowing for detection of overdose death spikes and identification of new patterns in contributing drugs in a shorter time window, which could be key as novel designer drugs emerge [35–37]. This could lead to faster mobilization of community stakeholders to implement harm reduction strategies, such as targeted naloxone distribution to communities.

An additional application of the ML method developed is its potential use as a data quality instrument. While many OD deaths are manually coded at NCHS [14], there is still the potential for coding errors to occur. Records that the classifier identifies as OD deaths but NCHS codes as non-OD deaths (false positives) can be reviewed by a medical examiner to determine if the case is truly a false positive or if the UCOD was coded incorrectly. This data quality process will capture OD deaths that previously were not identified and lead to more accurate, complete surveillance of the OD epidemic.

### Future directions

To further improve this model, additional feature engineering could be explored. This could include adding regular expression features to the model to identify patterns that frequently appear on OD DCs. Additionally, more n-gram features (such as non-adjacent bigrams) could be added to the model. Features that do not arise from NLP that use other fields on the DC (demographics, manner of death, place of death, etc.) could also be exploited in a future classifier. Part-of-speech tagging and dependency parsing, which creates a tree-like structure that explains the grammatical relationships between words in sentences [38], was considered for generating additional features for this analysis. Likely because the free-text present on DCs is often not grammatically correct, these features did not significantly increase the predictive power of the models. To build a simpler model they were excluded from the final analysis.

Deep neural networks, specifically convolutional neural networks, were considered for use in this task as they have been used for classifying free-text DCs [33, 34]. Due to the small amount of text present on DCs (some contain only two words) a traditional classification method was selected in our effort. Future research should examine deep learning methods (particularly the use of pre-trained word embeddings) and determine if they have higher performance for this task. In general, however, improving performance scores that are already in the high nineties (our F-score was 0.9695) will be a challenging endeavor that is worth further exploration. Deep learning should also be explored in the future for more complicated DC classification tasks, particularly multilabel classification of the substances that caused a drug OD death. An OD DC may contain no information about the substances causing the drug OD or list several substances involved in the death. A deep learning framework that can classify drug OD deaths and the substances causing the death from DC free-text would be a noteworthy extension to the methods developed in the present study. Since the completion of postmortem toxicology alone can delay the completion of the DC for weeks, the next step for improved timeliness in OD death surveillance is to expand the proposed ML models to work directly on medicolegal death investigation data, utilizing unstructured data from coroner and medical examiner case management systems (e.g., death scene investigation notes, autopsy reports, police reports, coroner notes) thus identifying likely drug OD cases before the DC is officially filed. As these reports typically contain much more free-text than DCs, deep learning models should be explored for this process as well.

### Strengths and limitations

The present study has several strengths. A thorough literature review indicates that this is the first study of its kind to our knowledge to describe a method for classifying OD deaths from free-text DCs using NLP and ML. The design of this experiment mimics how the model would be applied in a real-life use case scenario, with features engineered from previous year(s) data exploited to classify new data, giving the metrics on the test data validity for future use. This setup also adds to the difficulty of the task, as new substances that may be important in drug OD classification in a future year may not be present in the past year that the algorithm was trained on. Despite this, the ML models excluding the SCC field all achieved F-scores above 0.96.

Another strength of this study is the availability of data for our models to be tested on, further research to be performed, and similar algorithms to be developed. A major limitation of most ML applications is the large amounts of training data needed which coincides with the long, tedious process of labeling training data. Previous years coded DCs, however, are readily available for training at most state’s health departments. These DCs are ready to be used for training ML models without the typical initial requirement of labeling. This labeled data, while not publically available, can be requested from states’ OVS for research purposes. In addition, the NCHS operates the Research Data Center (RDC) to allow researchers access to restricted-use data. In 2019, the NCHS made available to researchers a Redacted Death Certificate Literal Text File (LTF) [39] that includes the cause-of-death text record for every U.S. resident death. The access to LTF will allow researchers to test our GitHub source code on DC records that come from all U.S. jurisdictions.

The study has some limitations. First, the models were trained on only Kentucky DCs, meaning that the model may not perform as well on data from other jurisdictions if certifiers use different words or phrases on their DCs. Kentucky, however, has a hybrid-coroner/medical examiner system with 120 county coroners and deputy coroners who certify deaths (per KRS 72.025) along with medical examiners who assist coroners in determining the cause and manner of deaths [40]. Physicians also certify natural deaths. Therefore, there is a diverse group of individuals certifying deaths in Kentucky, meaning the language on Kentucky DCs represents a range of medical backgrounds. Another limitation, inherent in ML, is the difficulty in diagnosing errors. The models described here have features numbering near the 10,000s—determining exactly what features are causing the model to incorrectly predict is inherently more difficult than for a simpler model with a small number of variables. A detailed error analysis of the SVM model excluding the SCC field is available in S2 Appendix.

## Conclusion

The present study compares three methods for identifying OD deaths from free-text DCs using NLP and several ML models as well as a simple rule-based method. Classifying OD deaths using free-text would substantially reduce the time it currently takes a surveillance systems to identify OD deaths, from several months to a few weeks. The described ML methods performed better than the rule-based methods on testing data, providing evidence that ML methods should be implemented in public health surveillance tasks, particularly for OD mortality surveillance. The programming code used to develop the model is publically available, which can facilitate further testing and development in other jurisdictions. Further research is needed to explore the potential for other causes of death to be classified using ML methods as well as additional exploration of ML, including deep learning, to improve drug-related surveillance.

## Supporting information

S1 Appendix Phrase list for rule-based method. (DOCX)

S2 Appendix Error analysis. (DOCX)
