# Supplementary material for: Enhancing timeliness of drug overdose mortality surveillance: A machine learning approach
Source: PLoS One. 2019 Oct 16;14(10):e0223318. doi: 10.1371/journal.pone.0223318 (PMC6795484; doi:10.1371/journal.pone.0223318)
Supplement: S1 Appendix — (DOCX) [file pone.0223318.s001.docx]

**S1 Appendix**

**Phrase list for the rule-based method**

1. Overdose
2. Polypharmacy
3. Drug intoxication
4. Multiple drug
5. Combined drug
6. Acute combined
7. Intoxication drug
8. Drug toxicity
9. Acute fentanyl
10. Fentanyl intoxication
11. Fentanyl toxicity
12. Heroin intoxication
13. Multidrug intoxication
14. Heroin toxicity
15. Acute intoxication
16. Combined effects
17. Toxic effects
18. Acute heroin
19. Multi drug
20. Multiple drugs
21. Illicit drugs
22. Abused fatal
23. Heroin fentanyl
24. Intoxication fentanyl
25. Fentanyl morphine
26. Intoxication methamphetamine
27. Acute multidrug
28. Intoxication heroin
29. Illicit drug
30. Acute methamphetamine
31. Drug fentanyl
32. Drugs including
33. Gabapentin drug
34. Methamphetamine intoxication
35. Intoxication overdose
36. Drug heroin
37. Including heroin
