# Supplementary material for: Enhancing timeliness of drug overdose mortality surveillance: A machine learning approach
Source: PLoS One. 2019 Oct 16;14(10):e0223318. doi: 10.1371/journal.pone.0223318 (PMC6795484; doi:10.1371/journal.pone.0223318)
Supplement: S2 Appendix — (DOCX) [file pone.0223318.s002.docx]

**S2 Appendix**

**Error analysis**

The false positives and false negatives of the SVM model excluding the SCC section were examined manually to determine if a post-processing step after the classifier is deployed could be used to improve classification. As Table 4 of the manuscript shows, this model had a total of 35 false positives and 21 false negatives on the test data. Of the 35 false positives, more than 10 appear to be data quality errors in the UCOD field on the DC records. These cases have free-text that points to the death being an OD death, but the text listed in the UCOD field is not an ICD-10 code.

Another category of the false positive cases are cases that appear to be OD deaths that were wrongly coded at NCHS. Of these cases, several of them mention the term “overdose” which likely lead the classifier to predict that these cases are ODs. Other cases in this category mention the decedent dying as a result of “drug intoxication” (listing a specific drug or indicating multiple drugs) or mention elevated levels of a substance in the decedent’s system. Interestingly, most of the cases in these categories list other complications, such as “asphyxiation due to drug overdose”, with NCHS then coding the cases as an asphyxiation death. However, if the OD was an event that directly caused the individual to asphyxiate, these deaths should perhaps be coded as OD deaths. This presents another use for this classifier—potentially identifying additional OD deaths that were miscoded at NCHS.

The majority of the other false positives are cases that involve deaths caused by chronic drug abuse (not an acute OD event) or an OD exacerbating some sort of condition or causing an injury that then leads to death. These cases include examples of an individual overdosing and then falling into a river and drowning, or an individual ingesting drugs and aggravating their existing chronic obstructive pulmonary disease. The presence of specific words, such as substances and terms like “intoxication”, likely lead to the classifier mistaking these for OD deaths.

Many of the 21 false negatives were cases with a small amount of text that contained substances that are rarely seen in OD deaths (such as acetaminophen) or contain misspellings. For example, one false negative misspells “toxicity” as “tocicity” and others misspell “drug” as “drue.” Employing an automated spell checker when preprocessing the text may fix some of these errors. The other false negatives are the opposite—well written text that is much longer than what typically appears on a DC. Many of these list that the decedent died from a combination of drugs and alcohol, so the presence of the word alcohol (or related terms such as ethanol) may signal the classifier that the death may be an alcohol related death and not a drug OD death, resulting in misclassification.
